# Drop-out in dual VET: why we should consider the drop-out direction when analysing drop-out

**DOI:** 10.1186/s40461-021-00127-x

**Published:** 2022-01-17

**Authors:** Maximilian Krötz, Viola Deutscher

**Affiliations:** Chair of Economic and Business Education–Competency Development and Training Quality, University of Mannheim, L4 1, 68161 Mannheim, Germany

**Keywords:** Vocational education and training, Drop-out, Intention, Training quality, Downward, Upward, Direction

## Abstract

Despite high drop-out rates from vocational education and training (VET) throughout most countries and a long research tradition on potential drop-out reasons, little is known about the effects exerted on drop-out intentions by the quality of training. Furthermore, only rarely do scholars distinguish between different drop-out directions, and systematic insights on possibly differing causes are scarce. This study explores the factors influencing four directions of drop-out intention (‘upwards’, ‘downwards’, ‘company change’, ‘occupation change’). Linear regression modelling is used to analyse survey data on the motivation, socio-demographic aspects and competency of 562 trainees as industrial management assistants in Germany and on how they perceived the training quality. The results show that different directions of drop-out intention stem from various factors, with training quality in general having the largest effect. Additionally, the findings indicate a two-tier-scheme of influence factors, ‘core’ and ‘direction-typical’ factors.

## Introduction

Drop-out rates in Vocational Education and Training (VET) are high throughout most countries,^1^ despite a long tradition in researching drop-out reasons (e.g. Barocci 1972; Grieger 1981; Weiß 1982). Two research factors could be contributing to the inability to substantially reduce those numbers. First, the effect of training quality on drop-out is underexplored, with most of the research focusing on learner factors (Böhn and Deutscher 2021). Second, scholars have rarely distinguished between different types of drop-out although the differing potential consequences of different dropout types are obvious. At the personal level, leaving vocational education completely, becoming unemployed or working without any qualification, constitutes a substantial cut in the individual’s biography, whereas continuing training in another company only results in a small, if any, loss of time (Autorengruppe Bildungsberichterstattung 2010, p. 109; Hensge 1988, p. 203; Weiß 1982, p. 283 ff.). A change in training occupation is associated with starting from scratch again while dropping out to attain a university degree could even increase future income. Impacts at the state level (e.g. tax revenue) or for society as a whole (e.g. shortage of skilled workers, expenses for the welfare-net) also differ depending on the drop-out directions. For training companies, however, a dropped-out trainee always causes increased costs (Autorengruppe Bildungsberichterstattung 2010; Deuer and Wild 2017; Hensen 2014; Schöngen 2003).

Not only are these two factors themselves interesting directions for future research, their interplay is also relevant as the different drop-out directions could stem from different causes. Therefore, greater knowledge about potential differences in the roots of distinct drop-out types could help practitioners to intervene more precisely and reduce drop-out rates in the future. We, thus, measure four types of drop-out intentions via a differentiated assessment (‘upwards’, ‘downwards’, ‘company change’ and ‘occupation change’). The objective of this study is to explore whether the widely applied general approach to drop-out intention (in the sense of an overall scale) is sufficient or whether a differentiation into different directions of drop-out intention leads to distinct results relevant for identifying potential causes for intentions to terminate training contracts prematurely. We examine this research question with data on the perceived training quality and competency of 562 individuals, training as industrial management assistants at the beginning and after the first year of training.

In the following, the underlying model of training quality, the concept of drop-out intention and a suitable measurement approach are presented. Moreover, an overview of the current state of research on the most frequent types of drop-out reasons is provided. In the main part, we introduce a differentiated assessment approach for drop-out directions consisting of four items and analyse whether the four directions of drop-out intention measure different facets of drop-out intention. We then regress training quality, competency and socio-demographic data on each direction of drop-out intention. Subsequently, results are presented and limitations and practical implications discussed.

## Premature terminations of contracts in VET

### In-company training quality

The theoretical basis for the meaning and content of training quality is provided by the quality model from Böhn and Deutscher (2019, p. 66) (Appendix Fig. 4). Developed from Tynjälä’s (2013) 3-p-model and Biggs (1999), the model distinguishes input, process and output dimensions of training quality. While the Input dimension includes all company and individual trainee characteristics existent prior to training (e.g. Work Climate, Learning Venue Cooperation and Demographic Factors), the process dimension comprises various training quality criteria that come into play in daily in-company training (Böhn and Deutscher 2019, p. 65 ff.). The Process dimension can be subdivided into three different areas (Work Tasks, Social Interaction and Educational Mediation), each covering three to five more detailed quality criteria (see Appendix Fig.4). Work Tasks comprises Overload, Variety of Tasks, Autonomy, Relevance of Tasks and Complexity of Tasks, which focus on covering different task characteristics of daily in-company training. Social Interaction and Educational Mediation reflect different areas of the interaction processes between trainees and trainers. Lastly, the Output dimension includes short- and long-term outcomes of vocational training and therefore comprises various aspects, e.g. Future Prospects and Career Aspirations or Operational Identity. This study, however, only focuses on drop-out intentions (Premature Termination of Contract) as an output variable. In line with the dynamic approach of the quality model, training quality is defined as the ‘subjectively perceived characteristics of training situations and processes that possibly affect target variables’ (Klotz et al. 2017, p. 3) such as drop-out intention.

### Reasons for dropping out

Much qualitative and quantitative research has been performed on why trainees drop out of VET, resulting in a long list of potential drop-out reasons. In a systematic overview, Böhn and Deutscher (in press) grouped drop-out causes into six different types: learner factors, professional factors, school factors, company factors, activity factors and context factors. The first four of those types belong to the Input dimension in the quality model (Appendix Fig. 4): learner factors (e.g. socioeconomic status), professional factors (e.g. expectations and decision making), school factors (e.g. school learning conditions) and company factors (e.g. work climate). The activity factors (e.g. requirements level and task characteristics) are part of the process dimension. The context factors include aspects regarding framework conditions (e.g. form or duration of training) and alternatives to training (e.g. finding a job without a qualification).

The overview showed that the research predominantly focused on Input factors, especially ‘learner factors’ (91% of analysed studies). Such inputs are already present, even before a trainee begins an apprenticeship. Surprisingly, aspects of the actual training process have rarely been considered. Therefore, the role of a vocational training’s process quality remains unclear, and only a few studies find effects on drop-out for process criteria (e.g. Cho et al. 2013; Hasler 2016; Krötz and Deutscher 2021; Negrini et al. 2016). Additionally, most studies ignore the direction of drop-out, i.e. the further course of education, if any, taken by trainees who terminate their original training contract. This omission leads to research pooling e.g. dropped-out trainees who aim to attain a university degree with those who become long-term unemployed. A few studies (e.g. Barocci 1972; Hasler 2016; Hensge 1988; Mischler 2014; Molgat et al. 2011; Schmid and Stalder 2012; Stalder and Schmidt 2006) consider the drop-out direction, but they do not systematically analyse different potential drop-out causes. Only Bessey and Backes-Gellner (2015, p. 548) differentiate between dropping-out and ‘upgrading’, as opposed to staying within the apprenticeship system. They find that the educational level, the financial situation and gender and ethnicity affect both groups differently. However, that study did not consider any aspects of training quality. In order to gain more knowledge about the drop-out causes during the training process, we believe considering both different drop-out directions and training quality criteria to be crucial.

### Operationalising drop-out and drop-out intention

The concept of drop-out, which is often measured via the premature termination of training contracts, constitutes a certain discontinuity in a VET path but does not necessarily imply a final withdrawal from VET. Training could be continued in another company or another occupation (CEDEFOP 2016; Schmid and Stalder 2012). Furthermore, drop-out figures do not generally indicate who (trainees or training companies) terminated a contract. When dealing with drop-out rates, the training sector and occupation also have to be considered, as differences are well documented (e.g. CEDEFOP 2016, p. 109; Hensen 2014, p. 5; Negrini et al. 2016, p. 363; Rohrbach-Schmid and Uhly 2015, p. 121). Also, most drop-outs seem to happen during the first year of training (Bundesinstitut für Berufsbildung 2020a; Cully and Curtain 2001; Lange 2020; Piening et al. 2010). Drop-out research scholars generally agree that the genesis of drop-out decisions covers a long period, rather than arising from a single event (e.g. Deuer 2003; Hensge 1988; Heublein and Wolter 2011) and that it is caused by multiple interrelated factors rather than a single, isolated reason (e.g. Ertelt 2003; Hensge 1984; Lamamra and Masdonati 2008; Rohrbach-Schmid and Uhly 2015). Therefore, in this study, drop-out is seen as a multifactorial process and operationalized as an output factor of training quality, in accordance with the quality model (Appendix Fig. 4).

**Fig. 1:**
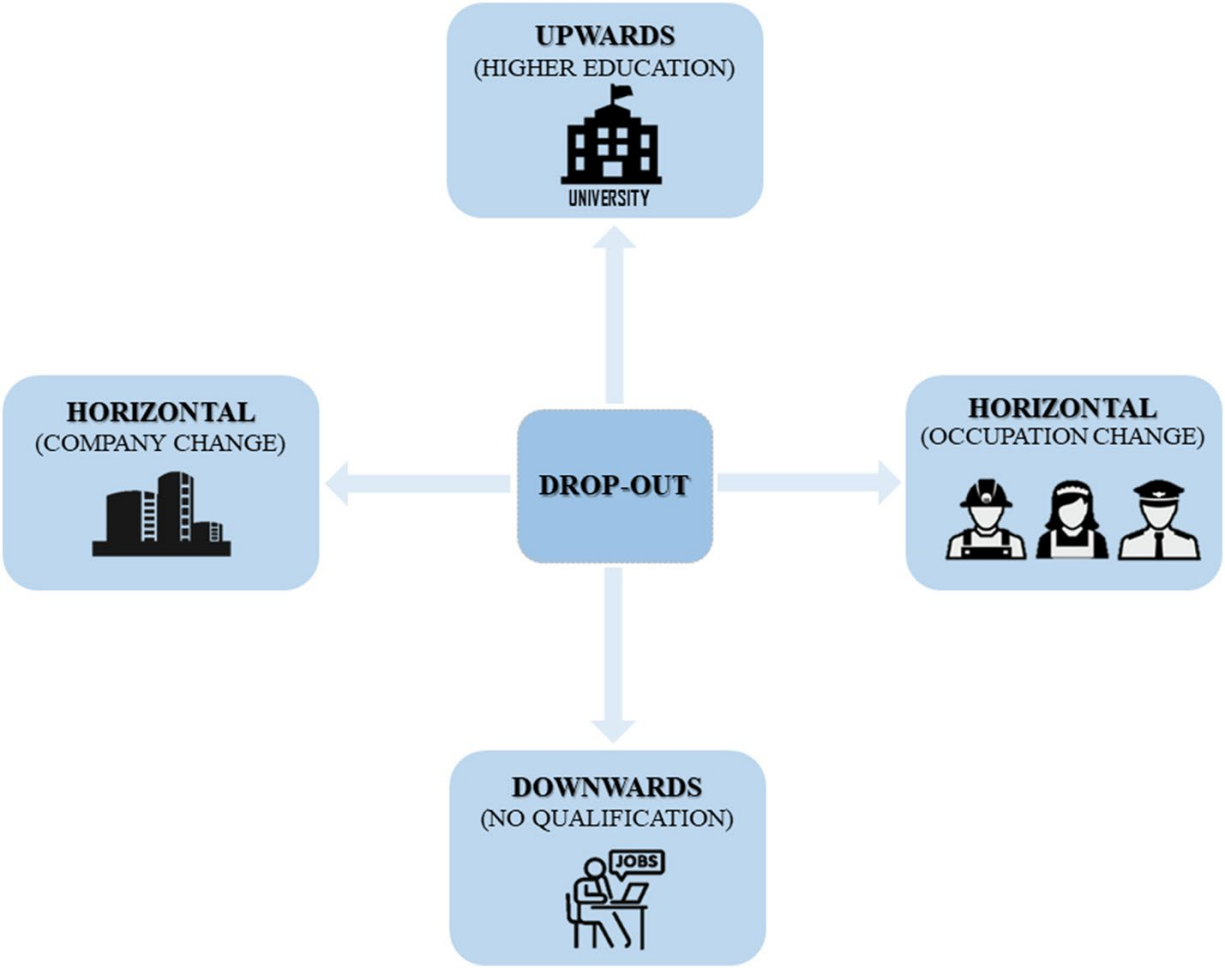
Differentiation of drop-out directions (as an extension of Feß 1995)

For the purpose of our research, we distinguish four different drop-out directions (see Fig. 1). Feß (1995, p. 29) differentiated three different types of drop-outs: upwards, horizontal and downwards. While dropping out upwards means attending further education outside of dual VET, e.g. attaining a university degree, a drop-out downwards represents the final withdrawal from VET, remaining unemployed or working in unskilled jobs. Lastly, a horizontal drop-out stands for vocational reorientation, such as starting an apprenticeship in a different occupation (Feß 1995; Faßmann 1998). We use this categorization but further differentiate between two types of horizontal drop-out: first, a horizontal change of training occupation and, second, a horizontal change of training company. A change in training company during an apprenticeship might allow the training process to continue relatively fluently and this path could have few negative consequences for an apprentice. Switching to a whole new occupation, in contrast, generally requires starting the apprenticeship from scratch. In both horizontal types of drop-out, trainees remain within the VET system.

Consequently, drop-out is defined as prematurely leaving the VET-system (upwards or downwards), the training company or the occupation as a result of the interplay of various input- and process-factors over a certain period of time, which are subjectively perceived and interpreted by each individual. Each of these four possible drop-out paths could conceivably be caused by different influencing factors. For instance, a trainee who wants to change the training company might be dissatisfied with certain quality aspects of the in-company training while someone who wants to switch the occupation might have had false expectations regarding vocational working life, and actual training quality might not be the central issue. Trainees who quit to go to university, might be under-challenged by the complexity of tasks or learning contents, whereas others who drop-out downwards might perceive these aspects inversely or have faced conflicts with colleagues or trainers. However, as mentioned above, quantitative studies on drop-out rarely consider these fundamental differences in drop-out directions on a methodological level. Therefore, almost no systematic findings on possible different causes are known to date.

An exception can be found in Weiß (1982, p. 286), who indicated an overrepresentation of trainees who dropped-out due to misbehaviour or for financial reasons in the group of downward drop-outs. Additionally, Mischler (2014, p. 47) showed on a descriptive level that a higher educational level increases the chance for a direct follow-up contract in the dual system or further higher education, whereas a higher age reduces the probability. Out of 175 trainees who terminated their contracts in a crafts business, 14.3% had no vocational perspectives after 4 to 12 weeks. Another 35.4% had only planned to start a new training, making it about 50% without a follow-up plan. Similar proportions (42–58%) are reported by e.g. Hasler (2016), Schmid and Stalder (2012) and Weiß (1982). These figures underline the great uncertainty a drop-out entails for young adults.^2^ The lack of systematic research on causes for different drop-out types is again surprising as findings on this question would provide a more solid fundament for more precise interventions and possibly preventing dropping out.

To gain insights into possible different causes of the four drop-out types, this study uses drop-out intention as a predictor of actual drop-out. Although used rarely, drop-out intention has been used as a practical alternative that bears relation to actual drop-out (see Bean and Metzner 1985; Deuer and Ertelt 2001, quoted from: Ertelt 2003; Deuer and Wild 2017; Gow et al. 2008; Quante-Brandt and Grabow 2008; Vallerand et al. 1997; Webb and Cotton 2018). While largely overestimating actual drop-outs, it entails substantial advantages for this research. Since dropping out is understood as a result of a process, measuring the intention to drop-out during training (and which variables interrelate with it) sharply increases insights into this process. This procedure may to some extent uncover the underlying influencing factors, which otherwise often become biased and abbreviated in retrospective approaches with actual dropouts (Aarkrog et al. 2018, p. 126; Rausch 2013, p. 56). Additionally, knowledge on drop-out intentions, as a sort of early alert signal, enables trainers and experts to intervene and prevent actual drop-outs and is therefore of highly practical use (Aarkrog et al. 2018; Deuer 2003). For each type of drop-out, a different item was used in the survey (see Survey instrument) according to our drop-out model in Fig. 1. Table 1 shows the four different questions used to operationalise drop-out intention in consideration of its direction (for descriptive results see Appendix Tables 9, 10).

**Table 1:** Operationalisation of drop-out intention considering four directions

Drop-out direction	Item
Upwards	I want to quit training to study at university (including dual university or university of applied sciences).
Horizontal (company)	I want to change my training company.
Horizontal (occupation)	I want to change my training occupation.
Downwards	I want to work without any training.

Measured on a five-level Likert scale (0 = strongly disagree; 4 = completely agree)

Concluding from the presented state of research, we expect that the four directions of drop-out intention in fact measure different facets and therefore should be analysed separately (H1). We then expect to find a significant relation between training quality and each drop-out intention (H2 a-d). However, in line with the findings of Bessey and Backes-Gellner (2015) and due to H1, we also expect each direction of drop-out intention to show partly different influencing factors (H3).

## Methodological procedure

### Data collection and sample

Data were collected as part of the project ‘Competence development through enculturation’ (KL 3076/2-1) funded by the German Research Foundation (DFG). The project involved surveys of industrial management assistants at the beginning of their training (T_0_: autumn of 2019) and after one year of training (T_1_: autumn of 2020). At T_1,_ trainees’ evaluations of training quality in companies and schools and their drop-out intentions were measured. At both points in time, competency tests were conducted, comparable to official final exams by the responsible Chamber of Industry and Commerce (IHK). The validated test instrument involved action-oriented tasks (e.g. writing a business mail, profitability and price calculations) embedded in an authentic, simulated company framework (see Appendix Fig. 5), measuring knowledge and practical skills (Deutscher and Winther 2018; Klotz 2015). Trainees’ socio-demographic background information and motivational-proxies were also collected at both stages. The first survey and test were conducted as a paper–pencil-format in randomly chosen vocational schools. The second data collection (in the same schools) was partly conducted online, due to the restrictions of the COVID-19 pandemic, but was kept identical in its content and presentation.

Both datasets were matched by an anonymous individual code that each trainee created. Because of the various socio-demographic variables considered, only cases where T_0_ and T_1_ data could be matched were considered in this study, leading to a potential sample of 610 trainees. To avoid biased results, all trainees who had already completed an apprenticeship were excluded from the analysis since their drop-out behaviour might differ considerably from trainees in their initial dual VET, given the security of already owning a qualification. The final sample amounted to 562 industrial management assistant trainees, 63.5% female. This proportion is near the typical distribution within the statistical population (latest three-year average 58.4% female, Bundesinstitut für Berufsbildung, 2020b). The average age at T_1_ was 20.6 years, ranging from 16 to 43 years, which is nearly identical to the average age (20.7 years) of the statistical population after one year of training (Bundesinstitut für Berufsbildung, 2020b). Most trainees only spoke German at home (77.8%), another 20.6% spoke German and additional languages, while less than 2% solely spoke foreign languages at home. Descriptive data regarding further sample characteristics is presented in Tables 7, 8 in the Appendix.

### Survey instrument

The survey on training quality mainly consisted of items and scales from the VET-learning quality inventory (VET-LQI) by Böhn and Deutscher (2021),^3^ which were supplemented with items on drop-out intention. In this survey instrument, all items and scales were formed on the basis of the quality model (Appendix Fig. 4). Therefore, all input- and process criteria included in the quality model (except for the area Framework) are used as training quality scales in the analysis. Additionally, scales on Professional Commitment, Teacher Competency and School Learning Content were included*.* All items and descriptive information for the scales are shown in the Appendix, Table 10. A satisfying Cronbach’s Alpha resulted for most of the 19 scales (0.73 ≤ α ≤ 0.91). Functional Involvement (0.67), Curriculum Orientation (0.65), Training Requirements and Ability Level (0.65) and Involvement in Occupational Expert Culture (0.63) showed slightly lower consistencies but, since they are important constructs in research on training quality, the scales were included in the analysis in order to represent training quality in a valid range (Schmitt 1996). All scales on training quality (as well as Desired Occupation) were measured on a five-level Likert scale (0 = strongly disagree; 4 = completely agree). Discriminant validity was checked by the intercorrelations of all quality scales (Appendix Table 11), which, if at all, correlated < 0.5. Only Social Involvement correlated slightly higher with Work Climate (0.555) and Feedback (0.523), which still satisfactorily indicates that the ‘social’ scales measure different quality constructs.

For socio-demographic, motivational and competency measures (see Appendix Tables 7, 8), most of the variables were collected in the first survey (T_0_). Only Age, the Aspired Final Grade and a self-assessment of Training Performance (in form of a grade) were used from the second survey. Also, Competency Scores at T_0_ and T_1_ were included.

### Analysis

For H1, descriptive data and correlations of the four drop-out intention items were analysed. Since the relation between training quality and the four different types of drop-out intention (H2) and also differences in their potential causes (H3) were being analysed, we conducted linear regression models and included socio-demographic and motivational variables, the competency scores and training quality scales as independent variables (see Appendix Table 10). For the complete sample, only one type of drop-out intention served as the dependent variable in each analysis. For missing values, pairwise exclusion was applied,^4^ still providing a sample size of 531 ≤ n ≤ 562 for most of the variables.

## Results

### Distinguishing four directions of drop-out intention

As Table 2 shows, the four directions of drop-out intention^5^ mostly correlate moderately (0.3 ≤ r ≤ 0.5; Cohen 1988, p. 79 f.). While the intentions to drop-out upwards and downwards show a small correlation coefficient (0.276), the intentions to drop-out upwards and change the occupation show a coefficient right on the edge of a medium effect (0.503). Therefore, we further analysed the group of trainees who clearly wanted to change their training occupation (responding with ≥ 3; n = 78; M = 3.59). Within this group, the average agreement for the intention to drop-out upwards increased (M = 1.71), as the constructs correlate to some extent, but stayed far below the intention to change the occupation. Furthermore, the ratio of the different intentions stayed the same, with company change being related relatively similarly (M = 1.96) and downwards showing the lowest relation (M = 1.29). Both analyses show that the four items sufficiently measure different directional intentions, confirming H1 and, therefore, implying the need to analyse the relation of training quality and drop-out intention in a differentiated approach.

**Table 2:** Intercorrelation of the four drop-out directions

Drop-out direction		Upwards	Horizontal (company)	Horizontal (occupation)	Downwards
Upwards	Correlation (Pearson)	1			
	Significance				
	N	547			
Horizontal (company)	Correlation (Pearson)	0.411**	1		
	Significance	0.000			
	N	546	549		
Horizontal (occupation)	Correlation (Pearson)	0.503**	0.478**	1	
	Significance	0.000	0,000		
	N	545	547	548	
Downwards	Correlation (Pearson)	0.276**	0.374**	0.387**	1
	Significance	0.000	0.000	0.000	
	N	543	546	545	546

**Correlation is significant at 0.01 (two-sided)

*Correlation is significant at 0.05 (two-sided)

### General overview of influencing factors on different drop-out directions

**Fig. 2:**
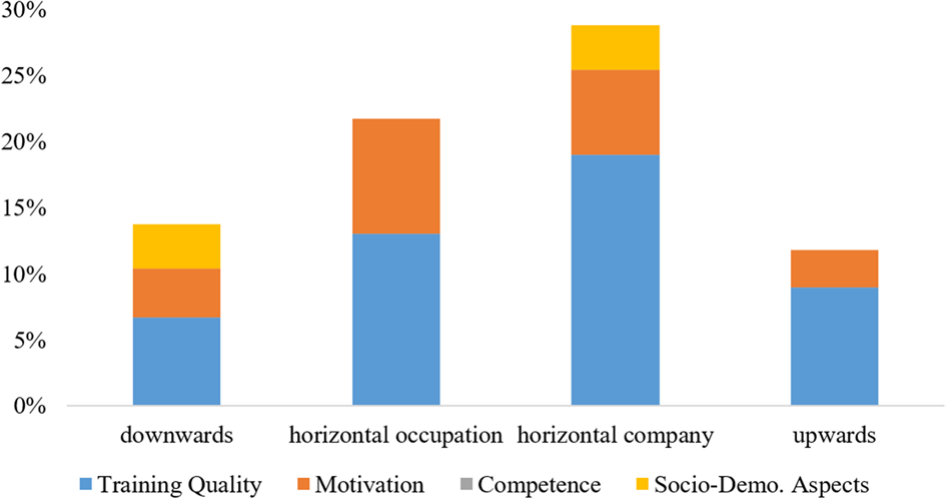
Influence factors on drop-out intention with respect to direction (visualized as R^2^ of global models)

As a first step, for each type of drop-out intention, a global model with four different blocks of variables was estimated. Block one contained basic socio-demographic variables, such as Age, Gender, Language (as dummies), the Educational Level, the corresponding Final Grade and a dummy for previously having Terminated Training elsewhere. The second block comprised the Aspired Final Grade in the current training (at T_0_ and T_1_), an item asking if it was the Desired Occupation before starting the training (0 = ‘strongly disagree’; 4 = ‘completely agree’) and the Professional Commitment scale, all as proxies for trainees’ overall motivation. The third block considered competency in the form of a subjective self-assessed Training Performance at T_1_ (as a grade) and the objective Competency Scores (T_0_ and T_1_). The final block included all 19 training quality scales plus an item (098) regarding Non-Training Tasks. Through this comprehensive block-wise procedure, it was possible to observe the changes in significance and R^2^, which we summarize in Fig. 2.^6^

Figure 2 visualizes how different areas contribute to explaining variance in the dependent variables.^7^ The graphical summary of which areas of influence factors exert a significant impact in the drop-out intention clearly shows the differences between the four types of drop-out intention. Here, the intentions to drop-out downwards and upwards could only be explained to a smaller extent by the independent variables while our survey-approach seems to be better suited for measurement of horizontal drop-out intentions. Particularly, training quality appears to play an immense role with respect to company change. Furthermore, motivational aspects seem to be involved in every type of drop-out direction, whereas socio-demographic aspects show mixed impacts. Competency, however, does not appear to be significant for any of the drop-out directions. At a first glance, the results shown in Fig. 2 seem to support our hypotheses 2 and 3. To examine the role of training quality and the potential differences between the directions in more detail, we formed narrow models out of the initial impressions gained, aiming at a maximum of variance explained, to find the most crucial predictors for each direction of drop-out intention.

### Predictors of downward drop-out intention

The most instructive model (Table 3), which includes only the relevant variables, comprises three training aspects and one socio-demographic aspect: A high Social Involvement and a good fit of the Training Requirements to the individual ability level reduce the intention to drop-out downwards.^8^ Also a lower Final Grade in the highest school leaving qualification (representing a better grade) is significantly related to lower drop-out intention. Conversely, the higher the Curriculum Orientation in training, the higher the intention to drop-out seems to be. This finding could indicate that a too stringent way of working along the curriculum may discourage some trainees. The model, however, only achieves a low level of variance explanation^9^ with an adjusted R^2^ of 0.086 (F[4, 423] = 11.06, p < 0.001).

**Table 3:** Narrow model 1: regression model on drop-out intention downwards

Predictors	B	SE	Beta	Sig.
(constant)	0.120	0.111		0.283
Social involvement	− 0.221	0.049	− 0.209	0.000
Training requirements and ability level	− 0.139	0.047	− 0.138	0.003
Final grade	0.101	0.042	0.111	0.018
Curriculum orientation	0.109	0.047	0.108	0.021

*B* regression coefficient, *SE*  standard error. R^2^ = 0.095, adjusted R^2^ = 0.086

### Predictors of horizontal drop-out intention (change of occupation)

Table 4 shows a narrower approach to the intention to change one’s training occupation (F[6, 421] = 18.69, p < 0.001). Responsible for a change in adjusted R^2^ of 0.136 alone, four training quality aspects appear to be especially important. A better Social Involvement and a more fitting level of Overload reduce the intention to drop-out. Again, a stronger Curriculum Orientation, but also higher Complexity of Tasks, significantly increase drop-out intentions. Moreover, the better the self-assessed Training Performance and the more the training corresponds to the Desired Occupation, the lower the intention to change one’s occupation.^10^ The model shows a medium-level variance explanation (adjusted R^2^ = 0.199).

**Table 4:** Narrow model 2: regression model on horizontal drop-out intention (occupation)

Predictors	B	SE	Beta	Sig.
(constant)	0.830	0.202		0.000
Training performance (T1)	0.162	0.055	0.129	0.004
Desired occupation	− 0.145	0.058	− 0.112	0.012
Social involvement	− 0.352	0.064	− 0.264	0.000
Overload	− 0.238	0.065	− 0.180	0.000
Curriculum orientation	0.127	0.056	0.099	0.025
Complexity of tasks	0.122	0.056	0.094	0.032

*B* regression coefficient, *SE*  standard error. R^2^ = 0.210, adjusted R^2^ = 0.199

### Predictors of horizontal drop-out intention (change of company)

In the stepwise selected and more instructive model shown in Table 5, only training quality aspects appear significant (F[5, 422] = 29.83, p < 0.001). The five criteria alone account for an adjusted R^2^ of 0.252, which is even higher than the results for the intention to change the occupation (Table 4). A higher quality, from the trainees’ perspective, regarding Feedback, Mentoring, Social Involvement, Overload and Non-Training Tasks lowers the intention to change the company during training. For the latter two aspects, reducing the workload and the number of tasks that do not contribute to training objectives appear important. With an R^2^ of 0.261 (adjusted R^2^ = 0.252), the model is right on the edge of a high variance explanation.

**Table 5:** Narrow model 3: regression model on horizontal drop-out intention (company)

Predictors	B	SE	Beta	Sig.
(constant)	1.126	0.161		0.000
Feedback	− 0.193	0.069	− 0.148	0.006
Mentoring	− 0.215	0.061	− 0.167	0.001
Overload	− 0.181	0.065	− 0.139	0.005
Non-training tasks	− 0.162	0.054	− 0.143	0.003
Social involvement	− 0.159	0.068	− 0.121	0.020

*B* regression coefficient, *SE*  standard error. R^2^ = 0.261, adjusted R^2^ = 0.252

### Predictors of upward drop-out intention

The final narrow model of upward drop-out intention (Table 6) includes the Educational Level and three training quality criteria (F[4, 423] = 11.32, p < 0.001). A higher Social Involvement and a better workload level reduce the drop-out intention significantly. Moreover, trainees who perceive the Complexity of Tasks to be high are more likely to drop-out upwards, which is also the case for trainees with a higher school leaving qualification. However, only 8.8% of variance in drop-out intention can be explained via the variables included in our study.

**Table 6:** Narrow model 4: regression model on drop-out intention upwards

Predictors	B	SE	Beta	Sig.
(constant)	0.079	0.158		0.616
Social involvement	− 0.206	0.059	− 0.179	0.001
Complexity of tasks	0.143	0.052	0.128	0.006
Overload	− 0.167	0.059	− 0.146	0.005
Educational level	0.157	0.066	0.111	0.017

*B* regression coefficient, *SE*  standard error. R^2^ = 0.097, adjusted R^2^ = 0.088

### Comparing the predictors of different drop-out directions

The results above can be summarized in that different directions of drop-out intentions are partly influenced by different factors. To verify the impressions, we compare the areas of influence factors based on the narrow models. Overall, the results look relatively identical to Fig. 2, where the horizontal drop-out intentions could be explained more extensively than the other intentions. Training quality is the area that shows, by far, the strongest relation to drop-out intentions. Variables stemming from other areas (Final Grade, Training Performance, Desired Occupation, Educational Level) play a minor role. A trainee’s intention to drop-out in order to change the training company can even be explained to a large extent (25.2%) by training quality alone. The findings underline the outstanding role of training quality for all directions of drop-out intention and, therefore, confirm H2 a-d.

For H3, several aspects indicate that, for drop-out research, it is worthwhile distinguishing between different types of drop-out intention. First of all, 12 different variables were identified as predominantly responsible for drop-out intention, with only two of them (Social Involvement and Overload) being significant for at least three (out of four) drop-out types. Both, Social Involvement and Overload could be working as a sort of ‘core’ influence on drop-out intentions for all types.^11^ However, in order to not ascribe Overload a core role, as it has not been fully identified, only Social Involvement is referred to as a core influence in the following.

Apart from Social Involvement, the downwards drop-out intention is mainly driven by too high Requirements, too stringent Curriculum Orientation and lower prior success or performance (in terms of a Final Grade). The intention to change the training occupation is mainly related to Training Performance, the degree to which trainees found their Desired Occupation, Overload, Complexity of Tasks and Curriculum Orientation. In contrast, the intention to change the training company is mainly related to bad Mentoring and little Feedback and to being charged with high workload (Overload) and Non-Training Tasks too often. Lastly, a drop-out upwards is mainly considered by trainees who perceive a high Overload, high Complexity of Tasks and who have a suitable Educational Level (as a necessary requirement to join a university) and therefore have the opportunity for an upward movement in their educational path.

**Fig. 3:**
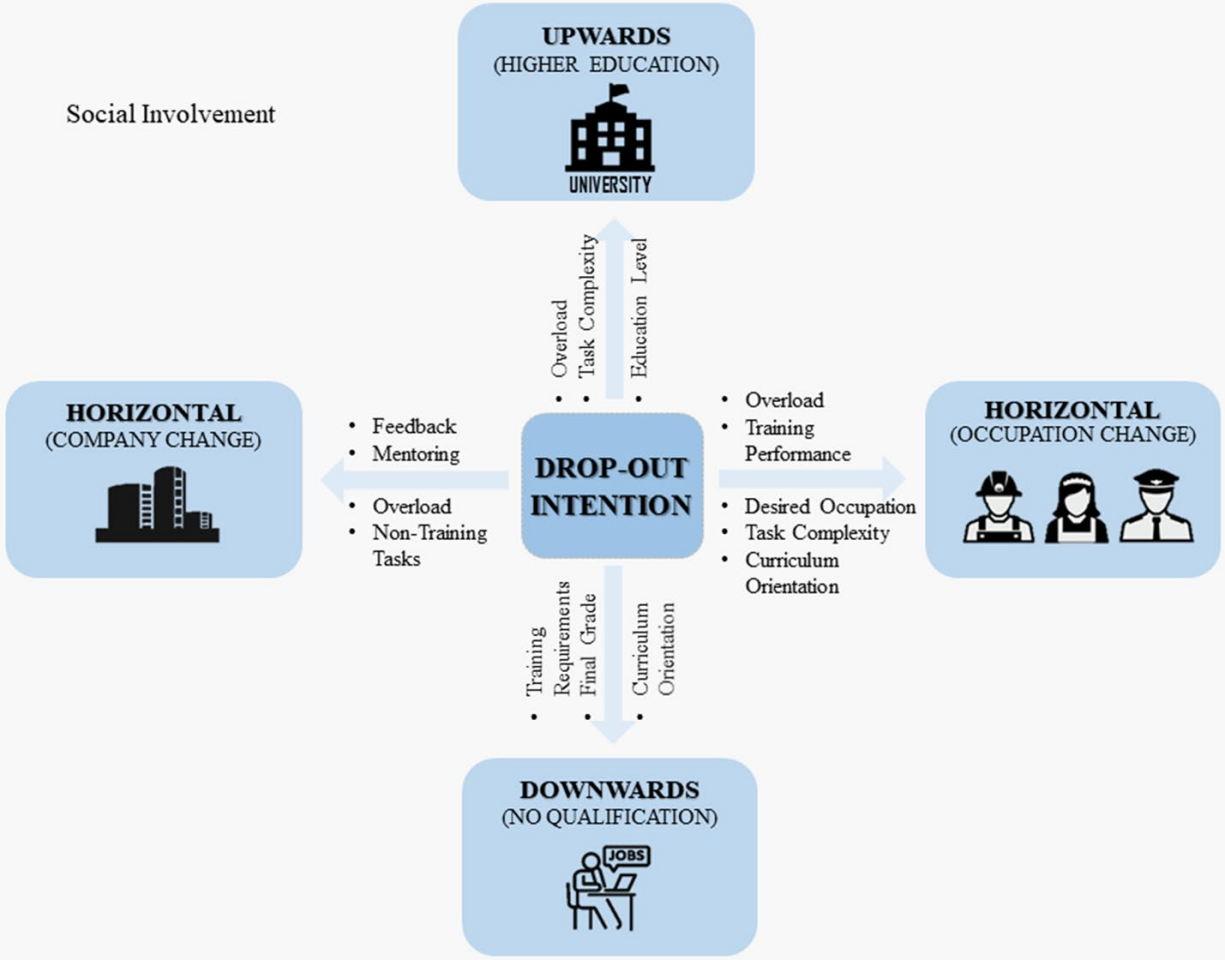
Two-tier-scheme of influence factors for different directions of drop-out intention

Aside from those factors, identified as crucial for drop-out intentions, 11 training quality aspects did not play a role for any drop-out direction. This finding could indicate a two-tier scheme (Fig. 3) with regard to the importance of different training quality aspects: (1) Social Involvement could be working as a core factor, and (2) one to four different quality criteria could be acting as ‘direction-typical’ factors. When we try to summarize the quality criteria on a more abstract level, the differences between the drop-out directions seem rooted in the extent to which Work Tasks (Workload, Non-Training Tasks, Complexity of Tasks) and Educational Mediation (Feedback, Mentoring, Curriculum Orientation, Training Requirements) is perceived (see Appendix Fig. 4). The more the Work Tasks are linked to the intention to drop-out, the more the occupation itself is consequently perceived by trainees as being suboptimal, leading to an intent to change occupation or to take a different path on a higher level (upwards). Contrastingly, changing the company or leaving the vocational path downwards seem to be more related to Educational Mediation. The insights gained allow the conclusion that H3 can be partly confirmed as there are several direction-typical factors and only few commonalities for the different drop-out intentions.

## Conclusion and discussion

Within this study, four directions of drop-out intention were analysed systematically and contrasted regarding their predictive factors for the first time. The analyses reveal diverse influencing factors for different directions of drop-out intention in vocational training. More precisely, the results, firstly, underline the complexity of the process, as stated in the literature (e.g. Ertelt 2003; Lamamra and Masdonati 2008; Rohrbach-Schimidt and Uhly 2015), with multiple factors being involved. The results, secondly, allow detailed insights into differences between various directions of drop-out intention and thereby shed light on the often referred to ‘black box’ of training. Training quality, especially social involvement during training, is key for all drop-out directions but particularly crucial regarding horizontal drop-out intentions. Upward and downward drop-out intention, however, can only be explained to a smaller extent by training quality. Here, also the educational level (including the Final Grade) plays a decisive role, corresponding to the results from Bessey and Backes-Gellner (2015).

Some of the findings should be interpreted with caution since the scales’ consistencies were not always satisfying. This is especially the case for Curriculum Orientation (Cronbach’s α = 0.65) and Training Requirements and Ability Level (Cronbach’s α = 0.65), which appeared significant in some models. However, the scales were kept in the analysis in order to secure a broad measurement of training quality in terms of construct validity. Moreover, it has to be noted that, with analysing data at T_1_, there is a certain amount of actual drop-out that had already taken place and could not be considered in any results. This difference could (partly) account for the relatively low drop-out intentions in the sample. Nevertheless, using the training quality measured at T_1_ was a conscious decision since the prior T_0_-survey was conducted very early, in some cases after 4 to 5 weeks of training (in which time a vocational school had also been attended), resulting in trainees who had little familiarity with the training companies’ qualities. A future design, where drop-out intention might be captured e.g. after 9–12 weeks, might further increase effect sizes due to more critical cases. With respect to the rather small explanatory power of the upward and downward models, other important aspects could be missing in our data, such as trainees’ general personal (life) situation or extrinsic motivation in terms of wage and prestige. Bessey and Backes-Gellner (2015) and Neuber‑Pohl (2021) showed that factors such as the financial situation or income prospects can be decisive for (downward) drop-out. Such variables could be analysed in greater depth regarding their influences on different drop-out directions in advanced future research designs.

With regard to the model of training quality (Böhn and Deutscher 2019), the findings confirm the processual structure, with drop-out intention being a result of the Input (e.g. educational level) and Process dimensions (training quality). Furthermore, the multidirectional approach to drop-out intention proved useful. Other classifications of drop-out types might be possible, as a vocational reorientation (horizontal directions) can also imply an upgrade with regard to the level of requirements or reputation. As a conclusion for future research, we recommend operationalising drop-out (intention) as a multidirectional concept as outlined in Fig. 1 if the complex causal nature of the concept is to be captured. As a conclusion for educational practice, a differentiation into different types of drop-out intention seems similarly important for training companies and trainers, especially if they are to intervene more precisely and prevent drop-outs in VET. For companies, a practical implication of the derived two-tier-scheme of influence categories is to lay focus on the social interaction with and involvement of trainees in all cases and then emphasize further direction-typical factors for the drop-out type where the individual company had experienced problems.

However, the findings for drop-out intentions cannot simply be transferred to real drop-outs, as, for instance, certain access barriers might impede the realisation of an intention to change occupation or attend university (e.g. due to qualification requirements). Additionally, not every drop-out has to be labelled negative, as a dissolution of a prior mismatch could lead to a more fitting career path in the future (Schmid and Stalder 2012). Nevertheless, many studies show that most dropped-out trainees remain for longer periods without a follow-up plan (Hasler 2016; Mischler 2014; Schmid and Stalder 2012; Weiß 1982). To impede the loss of time and the related costs, drop-out intention could serve as a useful tool in practice for gaining insights into the reasons behind drop-outs and as an early alert system, thereby helping to reduce drop-out in VET.

## Figures and Tables

**Fig. 4:**
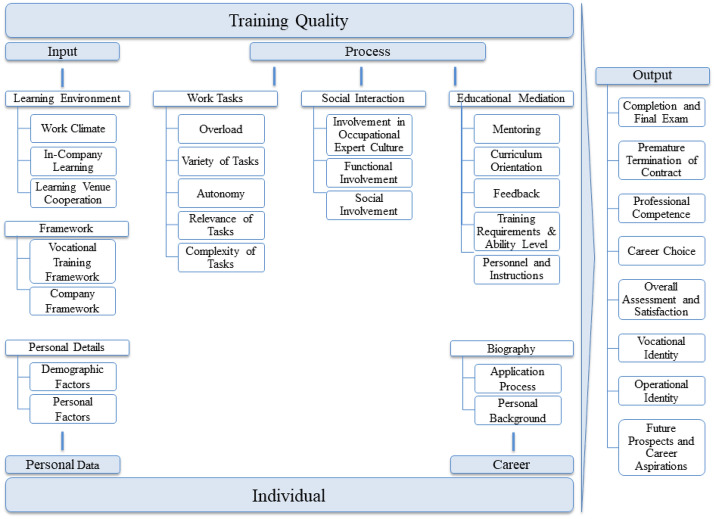
Operationalisation of training quality (Böhn and Deutscher 2019)

**Fig. 5:**
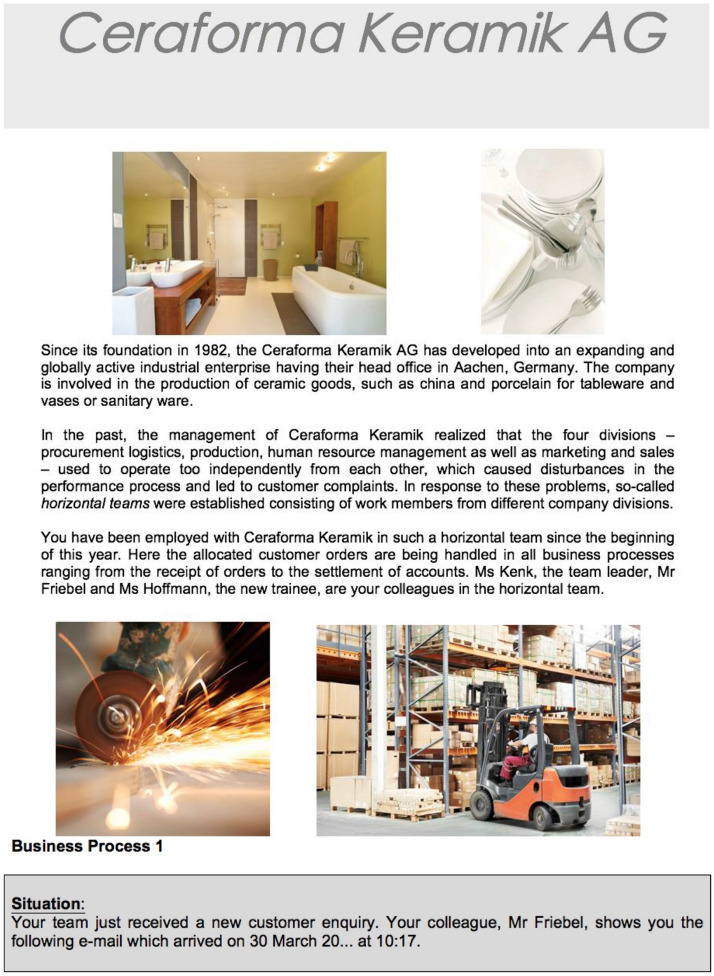
Exemplary company framework from the competency test (Deutscher and Winther 2018)

**Table 11:**
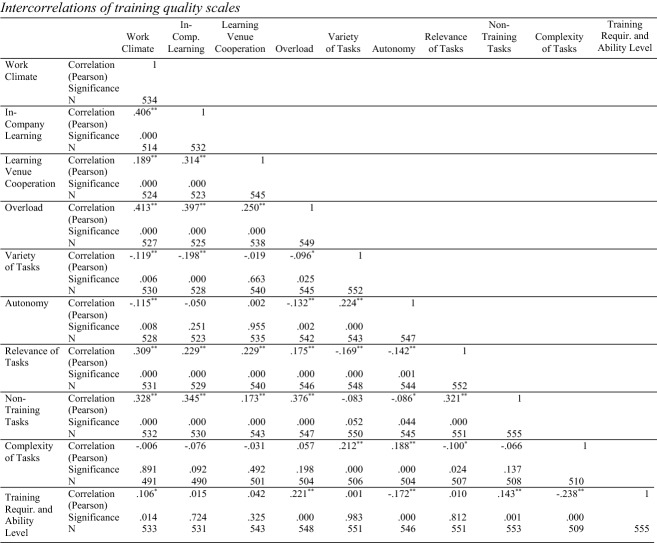

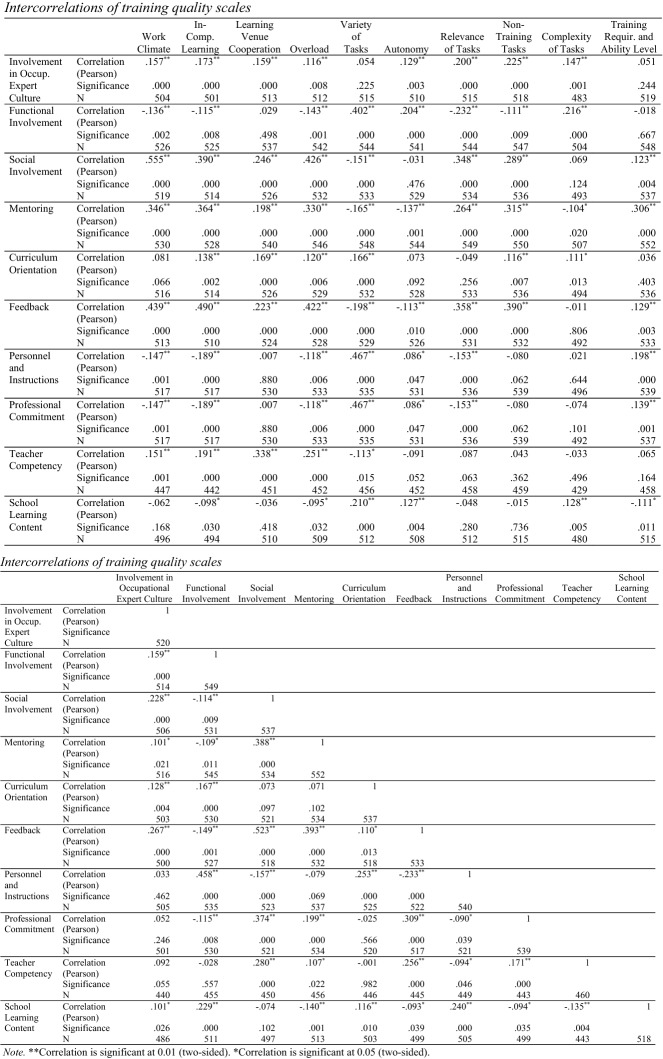
Intercorrelations of training quality scales

## Data Availability

The datasets used and/or analysed during the current study are available from the corresponding author on reasonable request.

## Appendix

See Figs. 4, 5 and Tables 7, 8, 9, 10 and 11.

**Table 7:** Personal background characteristics of trainees

Aspect	Coding	Frequency	Percentage	Valid percentage	Cumulated percentage
Gender n = 561	Female	356	63.3	63.5	63.5
	Male	205	36.5	36.5	100.0
Educational level (highest school leaving certificate) n = 562	Secondary school certificate (Mittlere Reife)	119	21.2	21.2	21.2
	Advanced technical college (Fachhochschulreife)	168	29.9	29.9	51.1
	General higher education certificate (allgemeine Hochschulreife/Abitur)	275	48.9	48.9	100.0
Grade (average grade in school leaving certificate) n = 555	1.0–1.5	39	6.9	7.0	7.0
	1.6–2.0	74	13.2	13.3	20.4
	2.1–2.5	175	31.1	31.5	51.9
	2.6–3.0	184	32.7	33.2	85.0
	3.1–3.5	77	13.7	13.9	98.9
	3.6–4.0	6	1.1	1.1	100.0
Training performance T_1_ (self-assessed grade) n = 548	1.0–1.5	62	11.0	11.3	11.3
	1.6–2.0	239	42.5	43.6	54.9
	2.1–2.5	160	28.5	29.2	84.1
	2.6–3.0	60	10.7	10.9	95.1
	3.1–3.5	23	4.1	4.2	99.3
	3.6–4.0	2	0.4	0.4	99.6
	> 4.0	2	0.4	0.4	100.0
Aspired final grade T_0_ n = 540	1.0–1.5	147	26.2	27.2	27.2
	1.6–2.0	285	50.7	52.8	80.0
	2.1–2.5	99	17.6	18.3	98.3
	2.6–3.0	8	1.4	1.5	99.8
Aspired final grade T_1_ n = 553	1.0–1.5	151	26.9	27.3	27.3
	1.6–2.0	245	43.6	44.3	71.6
	2.1–2.5	123	21.9	22.2	93.9
	2.6–3.0	29	5.2	5.2	99.1
	3.1–3.5	3	0.5	0.5	99.6
	3.6–4.0	1	0.2	0.2	99.8
	> 4.0	1	0.2	0.2	100.0
Language(s) (spoken at home) n = 559	Only German	435	77.4	77.8	77.8
	More than German	115	20.5	20.6	98.4
	Only other than German	9	1.6	1.6	100.0
Terminated Training before n = 561	No	524	93.2	93.4	93.4
	Yes	37	6.6	6.6	100.0

N maximum = 562

**Table 8:** Descriptive statistics on further trainee scales

Scale	N	Min	Max	M	SD
Age	561	16	43	20.57	2.504
Desired occupation*	558	0	4	3.01	0.994
Competency score T_0_	562	0	19	7.48	3.541
Competency score T_1_	536	0	24	10.92	4.779

N Maximum = 562. *Measured on a five-level Likert scale (0–4). Maximum Competency Score = 30

**Table 9:** Response frequency for different drop-out intentions

	Upwards		Company change		Occupation change		Downwards	
	Frequency	Percentage	Frequency	Percentage	Frequency	Percentage	Frequency	Percentage
0	468	85.6	423	77.0	427	77.9	481	88.1
1	15	2.7	29	5.3	22	4.0	15	2.7
2	12	2.2	18	3.3	21	3.8	4	0.7
3	17	3.1	37	6.7	32	5.8	19	3.5
4	35	6.4	42	7.7	46	8.4	27	4.9
Total	547	100.0	549	100.0	548	100.0	546	100.0

0 = strongly disagree, 1 = mostly disagree, 2 = partly agree, 3 = mostly agree, 4 = completely agree

**Table 10:** Item statistics

Scale	Item	Cronbach’s α (if item deleted)	Mean value	Standard deviation	Discriminatory power
*Personal factors*					
Professional commitment	008 I am motivated, no matter what kind of task I am confronted with	0.742	2.63	0.870	0.486
(α 0.77)	009 I am reliable, no matter what kind of task I am confronted with	0.741	3.58	0.602	0.465
	010 I am willing to put all my effort into my job	0.727	3.34	0.710	0.526
	xxx I finish every activity I have started	0.749	3.54	0.591	0.419
	xxx I am diligent at work	0.716	3.53	0.619	0.592
	xxx I am persevering at work	0.742	3.22	0.623	0.457
	xxx I work hard to achieve my professional goals	0.737	3.24	0.734	0.484
*Learning environment*					
Work climate	021 If necessary the employees in my company support each other	0.710	3.08	0.832	0.558
(α 0.76)	022 There is a personal atmosphere within my company	0.724	2.98	0.854	0.514
	023 There is a bad working atmosphere in my company. [R]	0.666	2.95	0.897	0.670
	024 There is strong competition between employees in my company. [R]	0.730	2.91	0.885	0.497
	025 Employees in my company are rigorously monitored and controlled. [R]	0.758	2.74	0.973	0.428
In-company learning	026 Workplace learning in my company is characterized by different teaching methods		1.86	1.053	0.723
(α 0.84)	027 Workplace learning in my company is characterized by the usage of different materials and media		2.11	1.052	0.723
Usefulness of learning venue cooperation	030 What I learn at vocational school is important for the daily work in my company	0.576	1.75	0.912	0.627
(α 0.74)	031 When managing work tasks in the company, I benefit from knowledge I accumulated during vocational school sessions	0.558	1.89	0.926	0.640
	033 The in-company vocational training and the vocational school are well coordinated	0.810	1.39	1.051	0.436
*Work tasks*					
Overload	045 In my company I feel under pressure of time at work. [*]	0.796	2.81	0.878	0.538
(α 0.82)	048 In my company others interfere with my work. [*]	0.814	3.29	0.783	0.433
	049 I have problems recharging my energy in my spare time after work. [*]	0.767	2.76	1.185	0.661
	050 Because of the daily demands in my company I feel totally exhausted, tired and drained. [*]	0.751	2.45	1.123	0.723
	051 I often think ‘I can’t go on any longer’. [*]	0.770	3.06	1.084	0.650
	xxx I have to do a lot of activities at once. [*]	0.808	2.19	1.080	0.480
Variety of tasks	052 In my company I deal with a variety of work tasks	0.831	2.45	0.899	0.424
(α 0.75)	053 In my company I work on new tasks every now and then	0.551	2.34	0.973	0.680
	054 In my company work tasks are highly diversified	0.572	2.40	1.019	0.662
Variety of tasks	052 In my company I deal with a variety of work tasks	0.831	2.45	0.899	0.424
(α 0.75)	053 In my company I work on new tasks every now and then	0.551	2.34	0.973	0.680
	054 In my company work tasks are highly diversified	0.572	2.40	1.019	0.662
Variety of tasks	052 In my company I deal with a variety of work tasks	0.831	2.45	0.899	0.424
(α 0.75)	053 In my company I work on new tasks every now and then	0.551	2.34	0.973	0.680
	054 In my company work tasks are highly diversified	0.572	2.40	1.019	0.662
Autonomy	056 In my company I am given flexibility in the timing of work tasks	0.795	2.35	0.956	0.376
(α 0.76)'	xxx In my company, I can make many decisions myself in my work	0.693	2.64	1.071	0.585
	057 In my company I am able to decide what means to take to reach a goal	0.662	2.52	1.030	0.639
Autonomy	056 In my company I am given flexibility in the timing of work tasks	0.795	2.35	0.956	0.376
(α 0.76)	xxx In my company, I can make many decisions myself in my work	0.693	2.64	1.071	0.585
	057 In my company I am able to decide what means to take to reach a goal	0.662	2.52	1.030	0.639
	058 In my company I am given an enormous amount of freedom in doing my job	0.653	2.50	1.014	0.656
Relevance of tasks	059 In my company I am given responsible tasks		2.71	0.955	0.661
(α 0.79)	060 In my company I work on ‘real tasks’		3.17	0.881	0.661
Non-training tasks	061 In my company I have to deal with several tasks that are not part of my vocational training program (e.g. make coffee, copying, etc.). [R]		2.87	1.131	
Complexity of tasks	063 In my company work tasks are characterized by considering a wide range of information	0.587	2.52	0.850	0.623
(α 0.74)	064 In my company work tasks are characterized by considering a wide range of objectives. [*]	0.671	2.63	0.899	0.551
	065 In my company work tasks are characterized by considering changes over time	0.703	2.54	0.886	0.523
Training requirements and ability level	067 In my company I am confronted with tasks that are too complicated. [*]		1.54	1.635	0.482
(α 0.65)	068 In my company I am confronted with tasks I am insufficiently trained and prepared for. [*]		1.66	1.610	0.482
*Social interaction*					
Involvement in occupational expert culture	072 I am involved in the improvement of work processes in my company	0.535	2.06	1.122	0.432
(α 0.63)	073 My ideas and proposals are considered in my company	0.529	2.05	1.058	0.437
	074 I am involved in the discussion of technical and professional issues in my company	0.522	1.88	1.137	0.441
Functional involvement	078 Basically, my work tasks play a crucial role for my department		2.33	0.991	0.511
(α 0.67)	079 I am well integrated into the operational working procedures		2.30	0.893	0.511
Social involvement	080 Employees in my company are interested in me	0.676	2.85	0.870	0.743
(α 0.80)	081 Employees in my company are interested in my private well-being	0.788	2.42	1.061	0.573
	083 Employees in my company seem disturbed by my presence. [R]	0.762	3.60	0.719	0.585
	084 Employees in my company ignore me. [R]	0.757	3.64	0.709	0.601
*Educational mediation*					
Mentoring	085 In my company nobody feels responsible for me. [R]		2.54	1.681	0.588
(α 0.74)	086 In my company I am completely left alone to myself. [R]		2.58	1.714	0.588
Curriculum orientation	089 I do know my in-company training plan		2.56	1.075	0.485
(α 0.65)	090 The arrangements of my in-company training plan are observed		2.56	1.137	0.485
Feedback	092 In my company good performances are praised	0.836	2.86	1.073	0.622
(α 0.86)	093 Normally I do know whether I perform work tasks satisfactorily or not	0.827	2.92	0.850	0.673
	094 I find it hard to figure out whether I perform work tasks satisfactorily or not. [R]	0.845	2.93	0.896	0.560
	095 The training personnel and my colleagues let me know whether I perform work tasks satisfactorily or not	0.814	2.77	0.969	0.732
	xxx The training personnel always give clear and convincing reasons for the assessment of my performance	0.819	2.61	0.973	0.706
	xxx The training personnel check my work results and give me factual feedback	0.843	2.65	0.972	0.578
Personnel and instructions	097 Those who train me on the job are able to answer difficult technical questions	0.853	2.15	0.926	0.745
(α 0.89)	098 Those who train me on the job can explain well	0.872	2.26	0.853	0.696
	99 There is a lot I can learn from those who train me on the job	0.849	2.13	0.940	0.756
	101 Those who train me on the job are technically competent	0.831	2.15	0.923	0.801
*Vocational school*					
Teacher competency	xxx My teachers explain well	0.895	2.36	0.872	0.724
(α 0.91)	xxx I like my teachers	0.891	2.64	0.858	0.756
	xxx My teachers want the best for me	0.886	2.62	0.911	0.789
	xxx My teachers always support me	0.884	2.54	0.894	0.801
	xxx I can ask my teachers anything	0.895	2.64	0.991	0.723
	xxx I feel supported by my teachers when I have personal problems as well	0.900	1.96	1.149	0.719
School learning content	xxx All of the important commercial foundations are taught in the classroom	0.736	2.32	0.830	0.349
(α 0.73)	xxx The school also teaches specialist knowledge that I need in the company	0.740	2.57	1.230	0.406
	xxx At school, my practical work from the company was consolidated through background information	0.631	2.61	1.018	0.632
	xxx In the course of learning in vocational school, I can network knowledge from different subjects	0.662	2.39	0.877	0.573
	xxx In class I understand how the content relates to operational practice	0.660	2.47	0.912	0.572
Output: drop-out intention					
Upwards	xxx I want to quit training to study at university (including dual university or university of applied sciences)		0.42	1.116	
Horizontal (company)	xxx I want to change my training company		0.63	1.276	
Horizontal (occupation)	xxx I want to change my training occupation		0.63	1.294	
Downwards	xxx I want to work without any training		0.34	1.023	

n = 562. [R] = reversed items. [*] = items reverse-scored for the analysis in order to facilitate understanding of the results. 4 represents maximum quality. Original response options: 0 = strongly disagree, 1 = mostly disagree, 2 = partly agree, 3 = mostly agree, 4 = completely agree

## Footnotes

1 Due to different calculation methods, drop-out rates in different countries are hardly comparable (see e.g. CEDEFOP 2016). However, OECD countries report rising drop-out rates (see also Böhn and Deutscher in press), varying between 18.7% in China (Yi et al. 2015) and 58.6% in Australia (NCVER 2020).

2 However, in some cases, higher satisfaction of the drop-outs themselves is possible when a prior mismatch was dissolved (Schmid and Stalder 2012, p. 127).

3 For a validation of this instrument see Böhn and Deutscher (2021) and Krötz and Deutscher (2021).

4 Using t-tests, we checked whether the group of non-responders on a certain variable differed significantly from the group of responders, with regard to any non-categorical variable: Only 1.4% of all cases showed significant differences between both groups.

5 For descriptive information of the four drop-out items see Tables 9, 10 in the Appendix.

6 Despite the number of variables considered, with the Variance Inflation Factor (VIF) remaining < 2.27, multicollinearity was no issue in the analysis (Hair et al. 2014).

7 Due to the large number of variables included in the global models, we consider changes in standard R^2^ in order to get a sense of the underlying processes. However, we avoid overemphasizing its meaning and interpret only the adjusted R^2^ of the narrow models in the further procedure.

8 All quality criteria were adjusted in the same direction, meaning a higher response represents higher training quality.

9 Classification of variance explanation according to Cohen (1988, p. 413 ff.): R^2^ ≥ .02 = small effect; R^2^ ≥ .13 = medium effect; R^2^ ≥ .26 = large effect of variance explanation.

10 An alternative model, including *Professional Commitment* instead of *Desired Occupation*, delivers the nearly same results, but ‘loses’ *Curriculum Orientation*. This indicates that both *Professional Commitment* and *Curriculum Orientation* might play a smaller role than the other variables in Table 3.

11 Drop-out intention downwards is the only type where *Overload* does not appear significant. However, the fit of *Training Requirements and Ability Level* could be the stronger requirements-related factor here, overlapping the aspect of *Overload*.
